# Matched-pair analysis of survival in patients with poorly differentiated versus well-differentiated glottic squamous cell carcinoma

**DOI:** 10.18632/oncotarget.14772

**Published:** 2017-01-20

**Authors:** Ping Chen, Wenbin Yu, Junwei Huang, Hongbo Xu, Guojun Li, Xiaohong Chen, Zhigang Huang

**Affiliations:** ^1^ Department of Otolaryngology Head and Neck Surgery, Beijing Tongren Hospital, Capital Medical University, Beijing, 100730, China; ^2^ Key Laboratory of Carcinogenesis and Translational Research (Ministry of Education/Beijing), Department of Head and Neck surgery, Peking University Cancer Hospital & Institute, Houston, TX 77030, USA; ^3^ Department of Head and Neck Surgery, Houston, TX 77030, USA; ^4^ Epidemiology, The University of Texas M. D. Anderson Cancer Center, Houston, TX 77030, USA

**Keywords:** differentiation, laryngeal carcinoma, prognosis, staging, survival

## Abstract

To compare survival outcomes between patients with poorly differentiated versus well-differentiated glottic squamous cell carcinoma (GSCC). Fifty-five patients with well-differentiated newly diagnosed GSCC were pair-matched to 55 patients with poorly differentiated GSCC according to age, sex, year of diagnosis, overall stage, treatment (surgery type, neck dissection, surgical margin, and chemoradiation), smoking, and alcohol use. Survival analysis was performed using Kaplan-Meier estimates, and matched-pair survival was estimated using the Cox proportional hazards regression model. Patients with well-differentiated GSCC had significantly better overall survival (OS) (*P* = 0.001), disease-specific survival (DSS) (*P* < 0.001), and disease-free survival (DFS) (*P* = 0.003) than patients with poorly differentiated GSCC. Moreover, matched-pair analysis indicated that increased differentiation was associated with a significantly reduced risk of overall death (HR, 0.18; 95% confidence interval [CI], 0.07–0.46), death owing to disease (HR, 0.16; 95% CI, 0.05–0.45), and disease recurrence (HR, 0.17; 95% CI, 0.07–0.41), and these risks were reduced approximately 4-fold, 3.7-fold, and 9-fold, respectively, after adjustment for cancer-associated variables. Survival differed significantly between the well-differentiated and poorly differentiated GSCC patients after adjustment for cancer prognosis-associated variables. Thus, identifying potential differences in the molecular characteristics between these two groups of patients would help to further stratify these patients and ensure appropriate individualized treatment decisions. Basing treatment strategies on the level of differentiation may improve survival.

## INTRODUCTION

Laryngeal squamous cell carcinoma (LSCC) is one of the most common head and neck cancers, and the incidence rate of LSCC ranks second among carcinomas of the respiratory system, after lung cancer [1–3]. LSCC has three subsites: supraglottic, glottic, and subglottic tumors [4]. Glottic squamous cell carcinoma (GSCC) originates from the true vocal cords, and approximately 51% of LSCCs are glottic primary tumors, of which 85% to 95% are GSCC [5]. Treatment of GSCC is determined according to the TNM classification system by the American Society of Clinical Oncology and the Scottish Intercollegiate Guidelines Network [6, 7]. Over the past several decades, the management of GSCC has substantively evolved to include the use of nonsurgical treatment methods, open conservation surgery, primary total laryngectomy, and endoscopic laser surgery, as well as concurrent chemoradiotherapy, but the long-term survival of GSCC patients has improved only moderately.

Studies regarding GSCC incidence, mortality, and prognosis often consider it as one entity, and historically, clinicians used TNM stage and smoking status as prognostic markers to guide treatment decisions for GSCC patients. However, the prognosis of these patients differs significantly between those with well-differentiated and poorly differentiated GSCC. For example, in our clinics, we have found that some patients with early TNM stage and poorly differentiated GSCC have a poor prognosis, whereas other patients with advanced TNM stage and well-differentiated GSCC have a good prognosis. Thus, patients with the same stage of GSCC may have different degrees of differentiation. It is our postulation that well-differentiated and poorly differentiated GSCC tumors may have different genetic components and thus different susceptibility to similar treatment, but it is not known whether these differences affect the responses of GSCC tumors to treatment.

Although both differentiation and stage are known to affect tumor aggressiveness, the effect of differentiation on the prognosis of GSCC patients at different stages is unclear. Furthermore, the prognosis of patients with GSCC is known to depend on a number of other clinical features, lifestyle factors, and epidemiologic variables, and these prognostic factors may further confound the survival analysis. Matched-pair analysis allows for the removal of many confounding factors and a more accurate comparison of survival. Thus, we were the first to perform a matched-pair analysis of patients with well-differentiated and poorly differentiated GSCC and compare survival to determine whether differentiation has any effect on the survival of GSCC patients at different stages. This study mainly aimed to provide new information to help make decisions about which treatment should be used to improve the individualized treatment of GSCC patients.

## RESULTS

### Demographic and clinical characteristics

Table 1 lists the matched characteristics of the two groups. There were no significant differences between the two groups regarding the matching variables. All patients received surgical treatment: total laryngectomy, partial laryngectomy, or CO_2_ laser microlaryngoscopic resection. Neck dissection included selective neck dissection, modified neck dissection, and radical neck dissection. A minority of patients (23.6%) in each group received postoperative radiotherapy or chemotherapy on the basis of their pathologic results.

**Table 1:** Matched patient characteristics

Matched Variables		WDG (*n* = 55)		PDG (*n* = 55)	
		No	%	No	%
Age (years)	Mean age	63.5 ± 1.6		61.6 ± 1.6	
	Median age	63.0		60.0	
	Range	44~84		38~85	
Sex	Male	54	98.2	54	98.2
	Female	1	1.8	1	1.8
Diagnosis time (year)	1997–2010	27	49.1	27	49.1
	2011–2014	28	50.9	28	50.9
Stage	I / II	33	60.0	33	60.0
	III / IV	22	40.0	22	40.0
Treatment					
Surgery type	CO_2_ laser	27	49.1	27	49. 1
	Partial/total laryngectomy	28	50.9	28	50.9
Neck dissection	No	31	56.4	31	56.4
	Yes	24	43.6	24	43.6
Surgical margin	Positive	6	10.9	6	10.9
	Negative	49	89.1	49	89.1
Postoperative	No	42	76.4	42	76.4
chemoradiation	Yes	13	23.6	13	23.6
Smoking	Ever	46	83.6	46	83.6
	Never	9	16.4	9	16.4
Alcohol	Ever	29	52.7	29	52.7
	Never	26	47.3	26	47.3

WDG, Well-differentiated group.

PDG, Poorly differentiated group.

As expected given the different degree of differentiation and disease stage, the patients with well-differentiated GSCC had better 3-year and 5-year OS rates than the patients with poorly differentiated GSCC (93.5% *vs* 68.8% for 3-year OS and 90.2% *vs* 50.9% for 5-year OS), and the patients with early disease had better 3-year and 5-year OS rates than the patients with advanced disease (96.3% *vs* 70.6% for 3-year OS and 92.9% *vs* 52.2% for 5-year OS). Similar differences were found for DSS and DFS between these two groups (data not shown). However, the study was designed to avoid selection biases by comparing groups tightly matched on the major prognostic, demographic, and treatment variables. Although this study was designed to compare whether patients with well-differentiated GSCC had significantly better survival than those with poorly differentiated GSCC, such analysis was seriously confounded by disease stage. We found that patients with well-differentiated GSCC and early disease stage had the best survival, and patients with poorly differentiated GSCC and advanced disease stage had the worst survival. However, patients with late disease stage and well-differentiated disease had significantly better survival than those with early disease stage and poorly differentiated disease. The 3-year and 5-year OS rates for patients with well-differentiated and stage I/II disease, well-differentiated and stage III/IV disease, poorly differentiated and stage I/II disease, and poorly differentiated and stage III/IV disease were 100% and 100%, 91.1% and 83.5%, 84.7% and 61.2%, and 41.2% and 33.0%, respectively.

### Survival analysis

**Figure 1:**
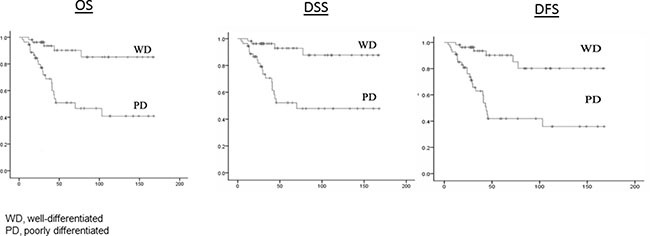
OS, DSS, and DFS by well-differentiated group and poorly differentiated group

**Figure 2:**
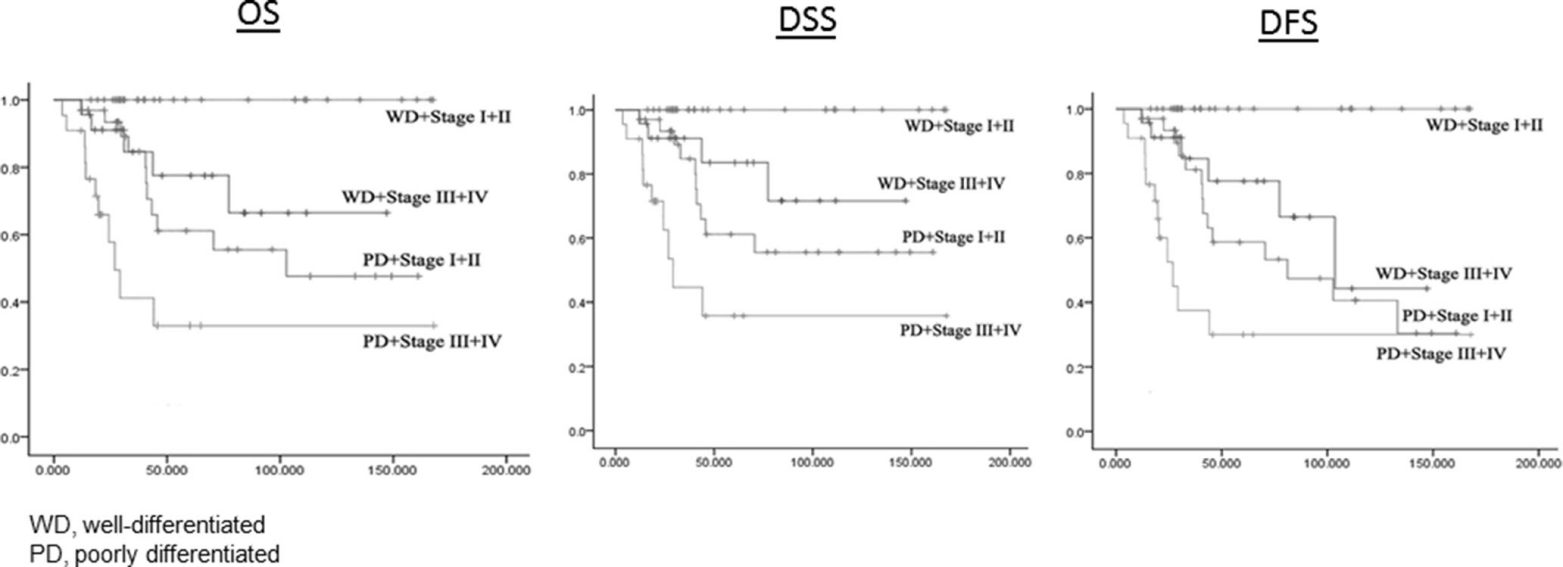
OS, DSS, and DFS by different differentiation and stage groups

Follow-up time ranged from 4.2 months to 170.6 months, with an average of 62.2 months (median, 44.0 months) for well-differentiated patients and 47.1 months (median, 29.0 months) for poorly differentiated patients. The follow-up time differed significantly between the two groups (*P* = 0.013). Patient outcomes for the two groups are listed in Table 2. For well-differentiated GSCC, 5 of the 55 patients died due to all causes, 3 of the 55 patients died owing to disease, and 6 of the 55 patients recurred, while for poorly differentiated GSCC, 22 of the 55 patients died due to all causes, 19 of the 55 patients died owing to disease, and 25 of the 55 patients recurred. Thus, patients with well-differentiated GSCC had much lower rates of death and recurrence than did patients with poorly differentiated GSCC. Figure 1 shows significant differences in the Kaplan-Meier OS, DSS, and DFS curves between well-differentiated and poorly differentiated GSCC (Log-rank, *P* = 0.001, *P* < 0.001, and *P* = 0.003, respectively). Figure 2 shows significant differences in OS, DSS, and DFS among the four groups of patients with different degrees of differentiation and disease stage (all log-rank, *P* < 0.001).

**Table 2:** Patient outcomes of follow-up by differentiation

Vital Status at Follow-up	WDG (*n* = 55)		PDG (*n* = 55)	
	No	%	No	%
Death, all causes				
No	50	90.9	33	60.0
Yes	5	9.1	22	40.0
Death, owing to disease				
No	52	94.5	36	65.5
Yes	3	5.5	19	34.5
Recurrence				
No	49	89.1	30	54.5
Yes	6	10.9	25	45.5

WDG, well-differentiated group; PDG, poorly differentiated group.

### Matched-pair analysis

In this matched-pair study, each pair was classified by the pattern of study events within the pair. Pairs in which each patient had the same events were considered concordant, and pairs in which one patient had an event and the other did not were considered discordant. There were three concordant pairs in which both the well- and poorly differentiated patients died, 14 discordant pairs in which the poorly differentiated patients died and the well-differentiated did not, and three discordant pairs in which the well-differentiated patients died and the poorly differentiated patients did not. Differentiation was associated with a significantly reduced risk of overall death (HR, 0.18; 95% CI, 0.07 to 0.46; *P* = .00). Multivariable analysis was performed for further adjustment with factors that significantly affect prognosis. The well-differentiated patients had an approximately 75% reduced risk of overall death compared with the poorly differentiated patients after multivariate adjustment (Table 3).

**Table 3:** Risk associated with differentiation

	Risk, WDG versus PDG			Risk after regression for cancer-associated variables*		
	HRs	*P*	95% CI	HRs	*P*	95% CI
Death, all causes	0.18	0.00	(0.07–0.46)	0.24	0.02	(0.07–0.79)
Death, owing to disease	0.16	0.001	(0.05–0.45)	0.27	0.06	(0.07–1.01)
Recurrence	0.17	0.00	(0.07–0.41)	0.11	0.00	(0.04–0.29)

* adjusted by cancer-associated variables: age, sex, stage, year of diagnosis, neck dissection, chemoradiation, surgical margin, surgery type, smoking, alcohol use.

WDG, well-differentiated group; PDG, poorly differentiated group; HR, hazard ratio; CI, confidence interval.

There were two concordant pairs in which both the well- and poorly differentiated patients died from disease, 12 discordant pairs in which the poorly differentiated patients died from disease and the well-differentiated patients did not, and two discordant pairs in which the well-differentiated patients died from disease and the poorly differentiated patients did not. There was a statistically significant reduction in risk of death owing to disease (HR, 0.16; 95% CI, 0.05–0.45; *P* = 0.001). Multivariate analysis was performed, fully adjusting for other important confounders, and the risk of death owing to disease was reduced approximately 75% in the well-differentiated patients (Table 3).

There were three concordant pairs in which both the well- and poorly differentiated patients had a disease recurrence, 16 discordant pairs in which the poorly differentiated patients had a disease recurrence and the well-differentiated did not, and five discordant pairs in which the well-differentiated patients had a disease recurrence and the poorly differentiated patients did not. There was a statistically significant decrease in risk of recurrence (HR, 0.17; 95% CI, 0.07–0.41; *P* = 0.00), and this risk among well-differentiated patients was reduced approximately 90% compared with the poorly differentiated patients after further adjustment for other important prognostic confounders (Table 3).

## DISCUSSION

No previous studies have reported matched-pair analyses evaluating the effect of differentiation on survival in patients with GSCC. We matched 55 well-differentiated patients to 55 poorly differentiated ones using the matching variables of age, sex, year of diagnosis, disease stage, smoking and alcohol use, and treatment. We found that differentiation was significantly associated with a lower risk of death and recurrence of GSCC. Furthermore, we found that patients with advanced-stage but well-differentiated GSCC had significantly better outcomes than those with early-stage but poorly differentiated disease. These findings suggest that differentiation affects the survival of patients with GSCC, particularly in patients with advanced disease. Both disease differentiation and stage should be taken into consideration before clinicians make decisions regarding treatment of GSCC.

In a study of 1252 cases of LSCC, the 5-year control rates of poorly differentiated and well-differentiated LSCC were 44% and 76%, respectively (*P* < 0.01) [8]. Poorly differentiated LSCC had a high rate of metastasis in cervical lymph nodes and a low survival rate [8]. Although it has been confirmed that poorly differentiated LSCC has high malignant and strong invasive ability, the American Society of Clinical Oncology and Scottish Intercollegiate Guidelines Network have not recommended a therapy plan according to pathology differentiation [6, 7]. In this study, we found that the 3-year and 5-year OS, DSS, and DFS rates in the poorly differentiated group were significantly lower than those in the well-differentiated group. To obviate the interference of operation types, we divided our patients into laser resection and open surgery groups and found that patients with poorly differentiated GSCC had a poorer prognosis than those with well-differentiated GSCC regardless of the type of surgery. One of the reasons for the poor survival in the poorly differentiated GSCC group could be the characteristics of severe malignancy, strong aggressive ability, and early transference associated with poorly differentiated GSCC. Therefore, we conclude that operation type might not affect the difference in survival between poorly differentiated and well-differentiated GSCC.

We found that among patients who didn't receive postoperative chemoradiation, patients in the poorly differentiated group had worse prognosis than those in the well-differentiated group. Postoperative chemoradiation could play an important role in the improvement of prognosis. Hence, surgery alone might not be suitable for GSCC patients with poorly differentiated tumors, even those with early-stage disease, and chemotherapy should be considered or even preferred.

Several factors, including age, sex, TNM staging, surgical margins, and lymphatic metastasis, may confound the prognosis of GSCC [9–12]. The death, disease recurrence, and metastasis rates of patients with poorly differentiated GSCC were significantly higher than those of patients with well-differentiated GSCC. The patients with poorly differentiated GSCC died due to local recurrence, distant metastasis, or both. Therefore, the main causes of the higher mortality rate of patients with poorly differentiated GSCC were postoperative recurrence and metastasis. To further control the rates of recurrence and metastasis, patients with poorly differentiated GSCC may have to be considered for comprehensive treatment regardless of their TNM stage.

Our findings suggest that well-differentiated and poorly differentiated GSCC could have different landscapes of genetic and epigenetic changes. It is therefore biologically reasonable to find differences in their clinical behavior, as molecular changes may result in more aggressive disease in poorly differentiated GSCC and lead to a worse prognosis.

At present, our treatment approaches have not substantively changed, but our findings suggest that more targeted therapeutic methods may help improve outcomes and optimize the patients’ quality of life. Some GSCC patients who present with more advanced disease and well-differentiated tumors have better survival than patients with early stage and poorly differentiated tumors. The current American Joint Commission on Cancer (AJCC)/Union for International Cancer Control (UICC) TNM staging system for GSCC was developed for differentiation-unrelated GSCC. A new staging system is needed to adequately predict outcomes of patients with differentiation-related GSCC.

Several limitations may exist in this study. It is likely that selection biases affecting either the patients referred to our institution or the patients recruited to this study could exist. We did not compare the outcomes of patients treated with surgery versus chemoradiation alone, and a prospective study is necessary to analyze different treatments for patients with poorly differentiated GSCC. Furthermore, GSCC patients were identified for enrollment without a strictly defined screening or follow-up regimen, and some patients had short follow-up times. Longer follow-up of these patients could help clinicians monitor the patients’ condition. Finally, we did not collect some other important data on smoking, such as intensity and duration for pack years smoked as well as family cancer history, while we are collecting such data and continuing to recruit such patients for our future larger studies.

In conclusion, GSCC patients with poorly differentiated tumors had worse OS, DSS, and DFS compared with those with well-differentiated tumors and equivalent age, sex, year of diagnosis, disease stage, smoking and alcohol use, and treatment in our hospital. These results are substantively confounded by disease stage. Although our findings suggest that the poorly and well-differentiated GSCC patients may have more fundamental differences, further studies are required to elucidate the molecular differences between these two groups and the effect of disease differentiation on survival. Therefore, more attention should be paid to the treatment of poorly differentiated GSCC. To improve survival and quality of life, when assessing the prognosis of patients with different tumor stages, the impact of differentiation is critical to making treatment decisions, particularly for poorly differentiated GSCC patients. Although confirmation of our findings in other patient populations is needed, we propose consideration of new GSCC categories as an alternative to the traditional GSCC categories.

## MATERIALS AND METHODS

### Study patient population

We reviewed the medical records of more than 1000 consecutive patients with newly diagnosed, previously untreated GSCC who were treated at Beijing Tongren Hospital (Beijing, China) from September 1997 to December 2013. All subjects signed an informed consent form; and the study protocol was approved by the institutional review board of Tongren Hospital. Only incident cases of pathologically confirmed poorly differentiated primary GSCC without distant metastasis or localized resectable lesions were enrolled. The exclusion criteria were as follows: (1) supraglottic or subglottic laryngeal carcinoma; (2) moderately or well-differentiated laryngeal cancer; (3) non-squamous cell carcinoma; (4) secondary onset; (5) recurrent disease; and (6) no initial treatment or only palliative care, or initial treatment at a different hospital.

Fifty-five patients with poorly differentiated GSCC were available for matching. We used the patient chart database, which contains epidemiologic and clinical information (e.g., age, sex, tobacco use, alcohol use, disease stage, treatment, degree of differentiation) to establish our matched-pair analysis. Well-differentiated GSCC was defined as tumor containing less cellular atypia and more keratin pearls, whereas poorly differentiated GSCC was defined as containing more cellular atypia and mitotic count, without keratin pearls.

After providing informed consent, all patients participating in this study had their demographic and exposure information retrospectively retrieved from their chart records. “Ever smokers” were those subjects who had smoked more than 100 cigarettes in their lifetime, whereas “never smokers” had smoked 100 or fewer. “Ever drinkers” were those individuals who drank alcoholic beverages at least once a week for more than 1 year, and the others were “never drinkers.” The patients’ medical records were also reviewed for primary site, clinical stage, treatment, differentiation, and outcome events. From this cohort, each poorly differentiated GSCC patient was matched with one well-differentiated GSCC patient. The matching variables were age (within ± 5 years), sex, year of diagnosis (1997–2010 *vs* 2011–2014), treatment received (surgery type, neck dissection, surgical margin, and postoperative chemoradiation), disease stage (stage I/II *vs* stage III/IV), smoking, and alcohol use. Fifty-five pairs of poorly differentiated and well-differentiated GSCC cases were matched. A control group of well-differentiated GSCC patients was selected at the same time. The matched-pair data were followed at a 1: 1 ratio. The pairing criteria were as follows: (1) age difference ≤ 5 years; (2) same sex; (3) diagnosis time < 1 year; (4) same TNM stage, clinical stage, therapeutic strategy (CO_2_ laser microlaryngoscopic resection, partial laryngectomy, or total laryngectomy, with/without radiotherapy; and (5) surgery as primary choice. All pathological diagnoses based on biopsies were confirmed by two pathologists.

### Patient follow-up

Patients were typically followed up and monitored throughout their treatment and post-treatment courses with regularly scheduled clinical and radiographic examinations. Patients were considered alive and free of disease recurrence if absence of disease was documented on the date of the last visit with the head and neck surgeon, head and neck radiation oncologist, or head and neck medical oncologist. There were no universal standards for imaging. Typically, patients had either routine serial imaging or follow-up imaging on the basis of symptoms or findings on physical examinations.

### Statistical analysis

SPSS statistical analysis software (version 17.0, SPSS, Inc., Chicago, IL, USA) was used for the statistical analyses. The primary outcome events of this study were overall deaths, deaths owing to disease, and recurrence. Time to recurrence was defined from the date of the end of treatment to the date of last follow-up or clinical detection of recurrent cancer (local, regional, or distant). Participants who were recurrence free or lost to follow-up were censored. Overall survival (OS) was defined as the time from first appointment to death from any cause or date of last follow-up. Disease-specific survival (DSS) was defined as the time from first appointment to death from disease or date of last follow-up. For both OS and DSS calculations, participants who were alive at the end of the study period or lost to follow-up were censored.

The differences in disease-free survival (DFS), DSS, and OS were compared between the well-differentiated and poorly differentiated GSCC patient groups using Kaplan-Meier estimates and the log-rank test for equality of survival curves. Matched survival analysis was completed using the Cox proportional hazards regression model. The assumption of proportionality was tested and met for the Cox proportional hazards analysis. Matching was accounted for in the Cox proportional hazards model by including a matching variable, which accounted for the matching in the analysis based on age, sex, year of diagnosis, treatment (surgery type, neck dissection, surgical margin, postoperative chemoradiation), disease stage, smoking, and alcohol use. Those factors, which were significantly different, were used as variables in the Cox proportional hazards model. The relative risk was obtained for each type of event between the well-differentiated and poorly differentiated GSCC patients. The power of the study to detect the relative risk obtained was based on the model of Dupont and Lummer [13]. Given our strict matching criteria for 55 matched pairs, the power of the study to detect a relative risk of 4 was 83%.

## Abbreviations

LSCC: laryngeal squamous cell carcinoma
GSCC: glottic squamous cell carcinoma
OS: overall survival
DSS: disease-specific survival
HR: hazard ratio
CI: confidence interval
